# Customized-Language Voice Survey on Mobile Devices for Text and Image Data Collection Among Ethnic Groups in Thailand: A Proof-of-Concept Study

**DOI:** 10.2196/mhealth.3058

**Published:** 2014-03-06

**Authors:** Kasemsak Jandee, Saranath Lawpoolsri, Pimsurang Taechaboonsermsak, Amnat Khamsiriwatchara, Peerawat Wansatid, Jaranit Kaewkungwal

**Affiliations:** ^1^Center of Excellence for Biomedical and Public Health Informatics (BIOPHICS)Mahidol UniversityBangkokThailand; ^2^Department of Tropical HygieneFaculty of Tropical MedicineMahidol UniversityBangkokThailand; ^3^Department of Family HealthFaculty of Public HealthMahidol UniversityBangkokThailand

**Keywords:** expanded program on immunization, EPI, ethnicity, mobile technology, smartphone questionnaire survey, voiced question

## Abstract

**Background:**

Public health surveys are often conducted using paper-based questionnaires. However, many problems are associated with this method, especially when collecting data among ethnic groups who speak a different language from the survey interviewer. The process can be time-consuming and there is the risk of missing important data due to incomplete surveys.

**Objective:**

This study was conducted as a proof-of-concept to develop a new electronic tool for data collection, and compare it with standard paper-based questionnaire surveys using the research setting of assessing Knowledge Attitude and Practice (KAP) toward the Expanded Program on Immunization (EPI) among 6 ethnic groups in Chiang Rai Province, Thailand. The two data collection methods were compared on data quality in terms of data completeness and time consumed in collecting the information. In addition, the initiative assessed the participants’ satisfaction toward the use of a smartphone customized-language voice-based questionnaire in terms of perceived ease of use and perceived usefulness.

**Methods:**

Following a cross-over design, all study participants were interviewed using two data collection methods after a one-week washout period. Questions in the paper-based questionnaires in Thai language were translated to each ethnic language by the interviewer/translator when interviewing the study participant. The customized-language voice-based questionnaires were programmed to a smartphone tablet in six, selectable dialect languages and used by the trained interviewer when approaching participants.

**Results:**

The study revealed positive data quality outcomes when using the smartphone, voice-based questionnaire survey compared with the paper-based questionnaire survey, both in terms of data completeness and time consumed in data collection process. Since the smartphone questionnaire survey was programmed to ask questions in sequence, no data was missing and there were no entry errors. Participants had positive attitudes toward answering the smartphone questionnaire; 69% (48/70) reported they understood the questions easily, 71% (50/70) found it convenient, and 66% (46/70) reported a reduced time in data collection. The smartphone data collection method was acceptable by both the interviewers and by the study participants of different ethnicities.

**Conclusions:**

To our knowledge, this is the first study showing that the application of specific features of mobile devices like smartphone tablets (including dropdown choices, capturing pictures, and voiced questions) can be successfully used for data collection. The mobile device can be effectively used for capturing photos of secondary data and collecting primary data with customized-language and voiced questionnaire survey. Using smartphone questionnaires can minimize or eliminate missing data and reduce the time consumed during the data collection process. Smartphone customized-language, voice-based questionnaires for data collection can be an alternative and better approach than standard translated paper-based questionnaires for public health surveys, especially when collecting data among ethnic and hard-to-reach groups residing in multilanguage-speaking settings.

## Introduction

The paper-based questionnaire survey is the most frequent method used to collect health data, especially in low- and middle-income countries, where there is normally limited resources and technology [1,2]. Despite the fact that this has been the standard data collection method for decades, there are still extensively reported problems with paper-based questionnaires, including frequent errors, high storage costs, and high double-data entry costs. Currently, electronic data collection methods have been developed with the aim of merging the processes of data collection and data entry [2]. Many devices, such as personal digital assistants (PDAs) and smartphones, have been adapted to serve as electronic devices for data collection, and all of these are increasingly being used in place of paper-based questionnaires [1,3]. These PDAs and smartphones have limitations, however, particularly when it comes to downloading data, because they require telephone signals or wireless networks. In addition, all data can be corrupted if PDAs or smartphones are misplaced or stolen, and data can be lost if they are damaged [4-6]. However, electronic devices for data collection offer the advantages of improved data quality and consistency through the use of automated validation procedures and data range checks. They can integrate different kinds of formats (images, texts, voice) which can easily be transferred over long distances through wireless networks. Moreover, electronic devices for data collection have many advantages over paper-based questionnaires. There is no need for data entry or multiple printings, (making them budget-friendly), they avoid problems arising from illegible writing, and they can use media during interviews. The disadvantage is that all collected data is digitally stored and there are no hard copies available if problems occur during data collection. Thus, the electronic devices need to be designed and developed carefully in order to minimize problems and enhance data collection speed over paper-based questionnaires. Previous studies using PDAs, smartphones, and Internet-based devices [4,5,7-9] have reported high response rates and short data-collection times.

In the context of poor research infrastructure and of increasing demands for large-scale health surveys, the affordability and availability of mobile phones and wireless networks create a viable alternative to traditional paper and pencil methods. A study conducted in a peri-urban settlement in South Africa to evaluate the use of mobile phones in surveys by using lay community health workers, reported that using mobile technology via mobile phones offer benefits over PDAs in term of data loss and uploading difficulties [5]. In contrast, another study compared the completeness of data collection using the paper method and electronic method using handheld computers in an office-based patient interview survey [4]. A better return rate was found on paper- vs electronic-based methods. This was due to technical difficulties experienced with electronic data collection, and stolen or lost handheld computers. However, only 0.04% of total items were missing on electronic surveys, whereas 3.5% were missing on paper ones. Although handheld computers produced more complete data than the paper method for return rate, they were not superior because of the large amount of missing data due to technical difficulties with the handheld computers [4]. In Uganda, hundreds of health workers have used PDAs provided by the Ugandan Health Information Network to collect health data in the field. They revealed that health workers report increased job satisfaction due to the greater efficiency and flexibility provided by the technology [10]. Additionally, a study from India points to users who would not return to the paper reporting, since the mobile phone reporting saved time [11].

Besides the reported advantages of using smartphones and PDAs during surveys in terms of data completeness and timeliness, the mobile-technology device may also offer an advantage when collecting textual and picture data in different ethnic dialect settings. Several major technological innovations contributing to survey-data collection have been developed including graphic user interfaces and multimedia computing. There were reports on the effectiveness of using auditory communication and audio-visual communication techniques as part of the data collection method, particularly in the multilingual settings [12,13]. The mobile technology device can be customized to speak the specific language for each group, and thus standardize data collection in the field. This study was conducted to develop and test new electronic data capture tools using mobile devices (ie, smartphone tablets) with customized-language, voice-based questionnaires for data collection among 6 ethnic groups who speak different languages. The use of mobile-survey data capture was compared with the classic paper-based questionnaire method for data collection about the knowledge, attitude, and practice of hill-tribe mothers/caretakers toward immunization of their children. The main objective of this study was to evaluate differences in data completeness, time consumed, participant’s satisfaction, and problems encountered during the two methods of data collection.

## Methods

### Study Sites

**Figure 1:**
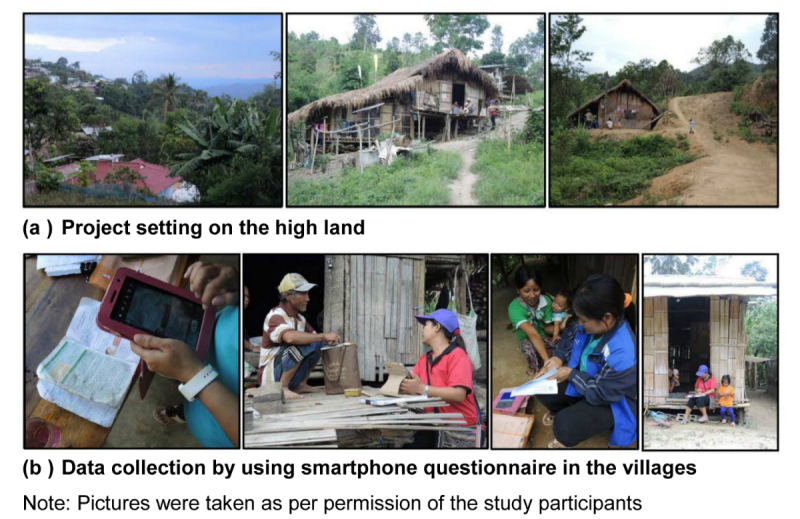
(a) Project setting and (b) data collection with smartphone questionnaire.

We conducted this study in 8 villages under the responsible areas of Wawi Sub-district Health Promoting Hospital (Mae Suai, Chiang Rai Province, Thailand). These areas are composed of 6 ethnic hill-tribal groups who speak different languages, have different cultures, beliefs, and lifestyles. These 6 hill tribes, include Akha, Karen, Mien/Yao, Lisu, Lahu, and Yunnan Chinese. Most of their spoken languages have no writing system. Figure 1a presents examples of study sites of these minority groups on the highland.

### Participants

A sample of 70 mothers with children older than 5 years in the study areas were recruited. The sampling frame was the names listed in the Wawi-database. The mothers registered in the Wawi database were selected by stratifying village locations and different ethnic groups. According to the routine activities for management of the Expanded Program on Immunization (EPI) as well as other health promotions under the Thailand Ministry of Public Health, each mother was approached (through a home visit) on a monthly basis by a designated Village Health Volunteer (VHV) to the approximately 10 allocated responsible households. Thus, the survey on EPI was conducted by 16 selected interviewers who are VHVs in the study areas. The interviewers were randomly selected and assigned to collect data from the randomly-selected hill-tribe mothers/caretakers.

### Mobile-Technology Initiatives

**Figure 2:**
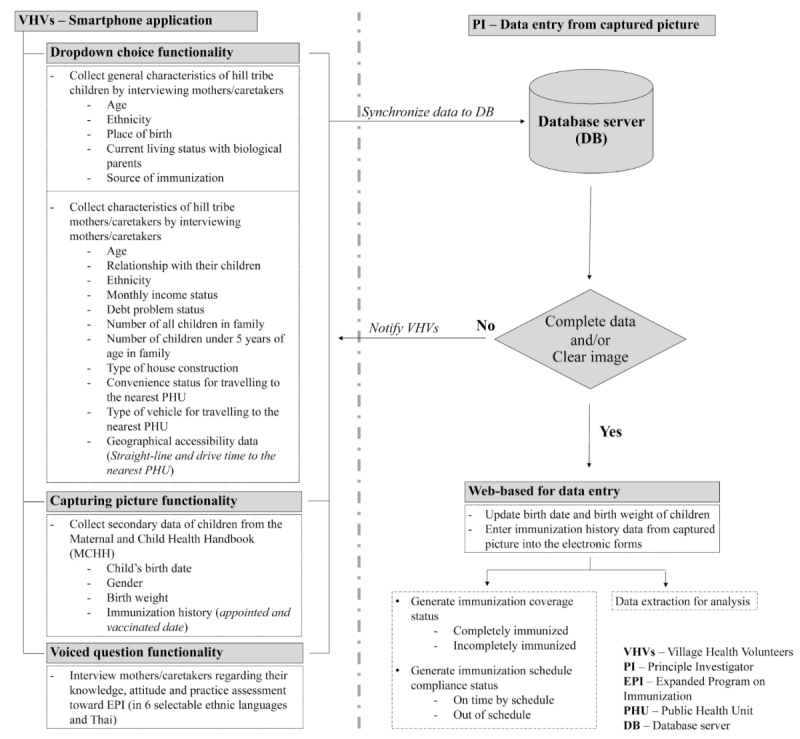
Concept and workflow of the smartphone questionnaire.

The hardware requirement for the mobile device to be used in the field is that the mobile-device hardware must run on the Java programming language. In terms of technical information about the mobile technology, the programming was done with Eclipse (an open source development tool) on Android. Sixteen smartphone tablet devices were distributed to the 16 VHVs. The concept and workflow of the smartphone questionnaires are summarized in Figure 2. Three data collection functionalities were programmed and installed on the mobile devices, including dropdown choices, picture capturing, and voiced questions. Data/information was collected offline and could be synchronized onto a server database when data collection was complete, and when there were telephone signals or wireless networks available. It should be noted that the personal information was treated confidentially within the system applications during the study period.

**Figure 3:**
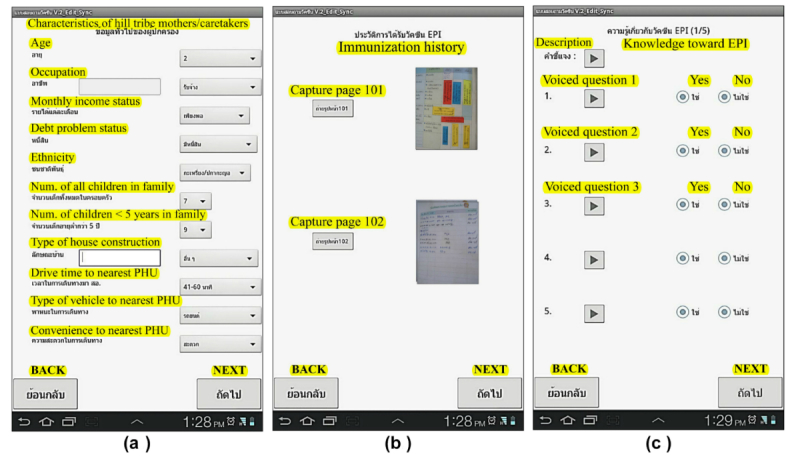
Feature of 3 functionalities on smartphone questionnaire: (a) dropdown choice, (b) picture capturing, and (c) voiced question.

Dropdown-choice functionality was developed and integrated into the mobile devices. The questions that were developed into dropdown-choice format, included sociodemographic parameters of hill-tribe mothers/caretakers and their children such as age, occupation, ethnicity, income and debt status, number of children in the family, type of house construction, drive time to the nearest hospital, vehicle and convenience for travelling to the responsible, district hospital in the area, child birth order, place of child birth, living with biological parents status, and source of vaccination. These questions with dropdown choices were asked at the beginning of the interview followed by other data collection techniques, specifically, picture capturing, and voiced questions. Respondents’ answers were automatically saved to the tablet’s memory. Figure 3a presents the feature for dropdown choices on the VHV’s smartphone questionnaire.

**Figure 4:**
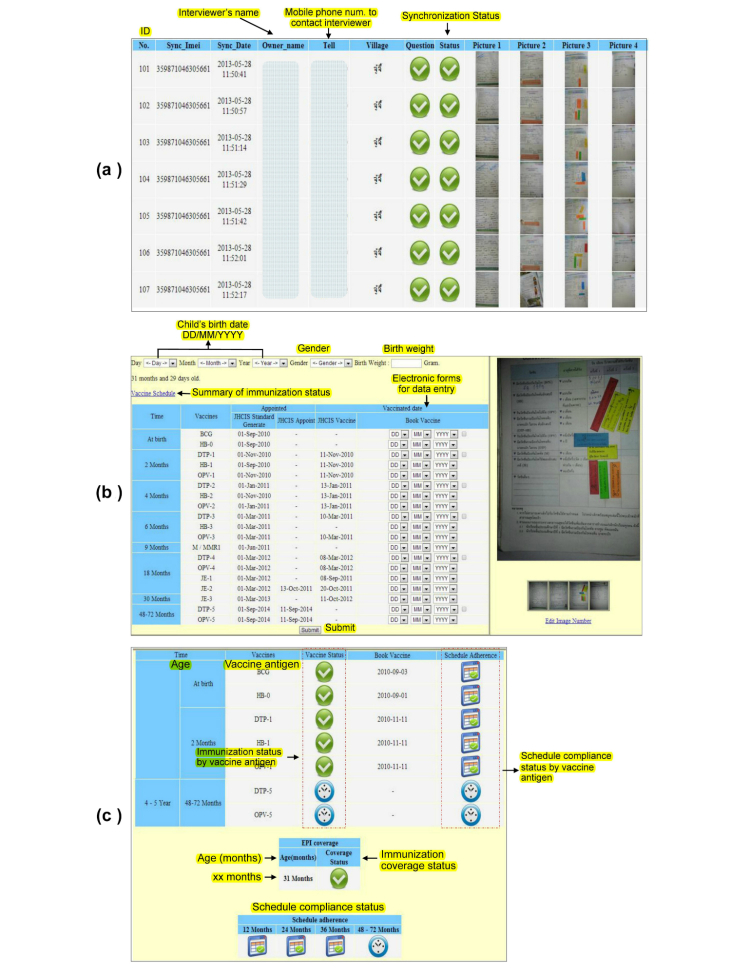
(a) Data monitoring on web-based, (b) immunization history data entry from captured picture, and (c) summary of individual immunization coverage and schedule compliance status.

Picture-capturing functionality is one of the methods for secondary data collection. This study had to collect data from the Maternal and Child Health Handbook (MCHH) regarding immunization history, scheduled vaccination date, and actual vaccinated date. Thus, capturing a picture of the history of immunizations over the years as shown on the MCHH booklet was incorporated into the questionnaire set. Collecting such information by an individual, self-reported interview or manually extracting data from the book would be quite cumbersome; thus, capturing a picture from the book would make it easier for interviewers. This could also eliminate data missing during the data collection process. The immunization history page was captured as a picture on the smartphone data-collection device by VHVs and saved automatically to its memory. If the picture was not clear, the VHV would repeat the picture-capturing process. Figure 3b presents the picture-capturing functionality for secondary data on the VHV’s smartphone. After all pictures were synchronized to the server, electronic forms were developed for data entry from the pictures. Immunization history data in the picture (scheduled and vaccinated date) was entered onto an electronic form. When all data was entered and submitted by the investigator, the immunization status (completely and incompletely immunized) and vaccine schedule compliance status (on time and out of schedule) were summarized and presented on the mobile device, which became a useful tool for VHVs to monitor the vaccine program of each child. Figure 4a-c presents the data entry from a captured picture and the summary statistics of individual immunization status, as well as their compliance to immunization schedule status.

Voiced-question functionality was purposively designed to collect data from 6 ethnicities who speak different languages, and where most of their languages have no writing system. The necessity of doing voiced questions on the mobile device arose from the fact that the VHVs cannot speak all six languages. This approach would also reduce the use of translators when performing data collection, and, in turn, lower mistranslations and standardize the question-asking process. The development of a voice questionnaire on smartphone tablets meant that the questionnaires were translated and incorporated onto the system screen into the 6 hill-tribe languages and Thai: Akha, Karen, Mien/Yao, Lisu, Lahu, Yunnan Chinese. This was done by local experts who are able to speak and read both Thai and his/her dialect languages. When performing data collection, the VHV would press the bullet button on screen for a question and the audio of the corresponding question would be spoken to the mothers/caretakers. The VHV then recorded the answers, all of which were multiple choice. In this study, the voiced questions in the smartphone tablet, included knowledge, attitude, and practice (KAP) of hill-tribe mothers/caretakers toward EPI. Figure 3c presents the feature for voice-question functionality regarding KAP toward EPI on VHV’s smartphone questionnaire.

### Training of Interviewers

All interviewers had experience using mobile technology (smartphone tablet) from the StatelessVac project, which was funded by a grant from the Bill & Melinda Gates Foundation through the Grand Challenges Explorations initiative [14]. The StatelessVac project was conducted in Chiang Rai Province from April, 2012 to April, 2013, and aimed to use mobile technology for enhancing routine EPI schedule reminders and behavioral change communication among ethnicity groups. Although the VHVs had practiced using mobile phone technology before, the use of mobile technology in this study was different from the StatelessVac. In this case, its purpose was data collection in surveys. Therefore, the 16 selected VHVs in this study received additional training on using smartphone, voice-based surveys and also to reinforce their data collection skills. They were trained for 2 days on the use of both smartphone tablets with voiced questions and paper-based questionnaire methods. Not only was training on using the mobile device provided, but the training also included communication skills and a real-life pilot practice interviews with hill-tribe mothers/caretakers using both data collection methods, explanation of questionnaires, translation of questions in each dialect languages, and practice to ask permission with informed consent from hill-tribe mothers/caretakers. All interviewers were trained individually to ensure that the two data collection methods were conducted correctly.

### Data Collection

**Figure 5:**
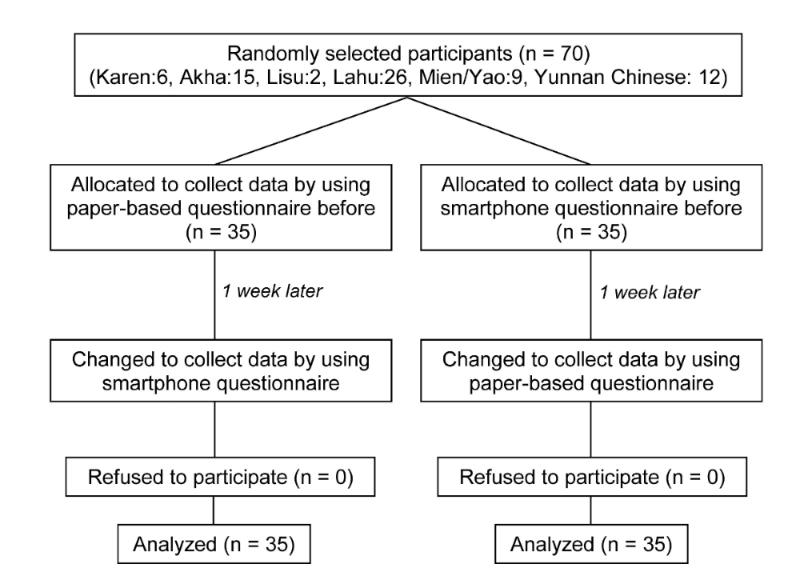
Workflow for data collection.

For the purpose of comparing the two data collection methods, customized-language, voice-based survey and the standard paper-based questionnaire regarding KAP, a cross-over design was adopted. The two groups of hill-tribe mothers/caretakers participants in this study were: (1) those surveyed by using a smartphone, voice-based then paper-based sequence, and (2) those using a paper-based then smartphone, voice-based sequence. The mothers/caretakers were randomly selected to the study groups with different sequences. The washout period between the two data collection methods was set at 1 week after the initial data collection method. Most studies with a time interval of the questionnaire administration for health-status instruments have ranged from 2 days to 2 weeks; however, a study investigating the 2-day and 2-week time durations reported no statistically significant differences in the test-retest reliabilities of the administered tests [15]. As it is generally recognized that a very short time interval might lead to the carryover effects, and with respect to the study population, a 1-week interval was selected to compromise the recollection bias in this study. The 16 VHVs who were interviewers in this study visited hill-tribe mothers/caretakers one by one in their routine job until the sample size of 70 was reached. Work flow for data collection is summarized in Figure 5. Figure 1b presents data collection using smartphone customized-language voice-based survey in the villages of the study area (note that all pictures presented in the figure were taken with permission from the villagers).

### Study Variables

The survey was composed of 16 sociodemographic items in typical multiple-choice fashion on the paper-based questionnaires, and with dropdown choices on the electronic questionnaires on smartphone, customized-language, voice-based applications. The picture capturing was on mobile devices only. There were 55 voiced questions, including 25 items on knowledge toward EPI vaccines, 15 items on attitude toward vaccination, and 15 items on practice for preparing or activities to vaccination. The outcomes for this particular study were not about analysis of vaccine coverage or on KAP issues (which will be described in another study) but on the comparisons of methodology outcomes from the two data collection methods. The main outcomes of interest were data quality composing of data completeness, time consumed, and participants’ satisfaction with the data collection methods. Data completeness and time consumed were based on repeated surveys with different methods from the same participant. The participants’ satisfaction toward the mobile technology survey was assessed using a simple Technology Acceptance Model (TAM) [16,17] in terms of perceived ease of use and perceived usefulness. Questions included how easy the survey had been to answer, whether the participant understood the questions clearly, their convenience during answering, and whether the electronic data collection had saved time. The satisfaction data toward using the smartphone, voice-based questionnaire survey method was collected from mothers/caretakers after the data collection process was finished.

### Ethical Considerations

This study was a part of the project “Assessment of Expanded Program on Immunization Coverage and its Determinants among Hill Tribe Children, Wawi Sub-District, Mae Suai, Chiang Rai Province, Thailand” (awaiting publication). The project was reviewed and approved by the ethics committee of the Faculty of Tropical Medicine, Mahidol University (Thailand). This study involves vulnerable research participants belonging to ethnic groups in Chiang Rai province, Thailand. All participants were informed about all details regarding the study, and asked to sign an informed consent form for participating by using their dialect language. The document was translated by VHVs.

There was no identification of name and family name of the respondents on the case record forms. The individual information was kept completely confidential to the researcher during data collection and analysis. The respondents were able to stop giving an interview at any time and did not need to give reason for the withdrawal of their consent.

## Results

### Picture Capturing of Data

During the study period, 70 hill-tribe mothers/caretakers were randomly selected from 363 mothers in the EPI-coverage assessment project. As part of the initiative of data collection on smartphone survey, the function of picture capturing was developed and used to collect the history of immunizations over the years in the MCHH booklet. Pictures of all pages with EPI recorded on the booklet of each child were taken and then directly transcribed into database. The data entry screen was designed corresponding to all data fields on the booklet pages, and thus all data were manually entered onto the system for further analysis on EPI coverage. The results of EPI coverage were beyond the purpose of this study.

### Completeness of Data

To assess the effectiveness of the two data collection methods, 35 study participants were randomly assigned to one of the two study arms in the cross-over of the standard paper-based questionnaire survey and smartphone, voice-based survey methods. The questions captured on smartphone tablets were programmed to collect and store answers in a sequential manner with no skip pattern, and thus no data entry errors occurred after data synchronization. Hence, the use of smartphones with the voiced-question method produced complete answers significantly different from the answers obtained from the use of the paper-based questionnaire method. In the paper-based questionnaire method, 69% (48/70) of hill-tribe mothers/caretakers answered all questions completely (as opposed to the 70/70, 100% of smartphone voice-based method). Out of the 55 items on KAP, the number of questions that had incomplete answers on the paper-based survey method ranged from one item (among 17/70, 24% of respondents) to six Items (1/70, 1% of respondents). As shown in Table 1, the separate group analysis reveals that the sequence of data collection method (Group 1: paper before voice, and Group 2: voice before paper) had certain effect on data completeness. The incomplete answers of the paper-based method of Group 1 still ranged from one item (10/35, 29% of respondents) to six items (1/35, 3% of respondents), but for Group 2 only one item (7/35, 20% of respondents) was incomplete**.**

**Table 1:** Data completeness between paper-based and smartphone voiced questionnaire survey methods.

Variables		Paper QNN		Voiced QNN		*P*
		_n_	%	_n_	%	
**Groups 1 and 2 (n=70)**						<.001^a^
	Complete answer	48	69	70	100	
	Incomplete answer	22	32	-	-	
	1	17	24			
	4	2	3			
	5	2	3			
	6	1	1			
	Range (minimum–maximum)	6(49-55)		-		
**Group 1: Paper-voiced (n=35)**						
	Complete answer	20	57	35	100	<.001^a^
	Incomplete answer	15	43	-	-	
	1	10	29			
	4	2	6			
	5	2	6			
	6	1	3			
**Group 2: Voiced-paper (n=35)**						
	Complete answer	28	80	35	100	.016^a^
	Incomplete answer	7	20	-	-	
	1	7	20			

^a^McNemar Test used

### Time Consumed During Data Collection

During the data collection process with both the smartphone, voice-based and paper-based questionnaire methods, the time consumed was measured. Time consumed was calculated in minutes from the time the first question was asked until the last KAP question was answered. Table 2 shows the mean duration of time consumed for data collection and the frequency of participants who spent different time slots to answer all questions. The mean duration of time spent on paper-based data collection was 32.39 minutes, compared with 22.51 minutes for a smartphone, voice-based survey. Of all participants, 37% (26/70) and 49% (34/70) spent 0-15 and 16-30 minutes, respectively, for data collection with the smartphone, voice-based method while 23% (16/70) and 43% (30/70) for the paper-based questionnaire method. The time consumed was significantly different between the two methods; the smartphone, voice-based questionnaire method was significantly faster than the paper-based method (*P*<.001). When analyzed separately, for the two sequences of data collection methods, (paper–voice and voice–paper groups), significantly shorter times for the smartphone, voice-based method were still observed.

**Table 2:** Time consumed between paper-based and smartphone voiced questionnaire survey methods.

Variables				Paper QNN		Voiced QNN		*P*
				_n_	%	_n_	%	
**Groups 1 and 2 (n=70)**								
	**Time consumed (minutes)**							<.001^a^
			0-15	16	23	26	37	
			16-30	30	43	34	49	
			31-45	7	10	-	-	
			46-60	14	20	10	14	
			61 minutes over	3	4	-	-	
	Mean			32.39		22.51		
	Range (minimum–maximum)			84(6-90)		56(4-60)		
**Group 1: Paper-voiced (n=35)**								
	**Time consumed (minutes)**							<.001^a^
		0-15		8	23	14	40	
		16-30		11	31	16	46	
		31-45		4	11	-	-	
		46-60		9	26	5	14	
		61 minutes over		3	9	-	-	
**Group 2: Voiced-paper (n=35)**								
	**Time consumed (minutes)**							.001^a^
		0-15		8	23	12	34	
		16-30		19	54	18	51	
		31-45		3	9	-	-	
		46-60		5	14	5	14	
		61 minutes over		-	-	-	-	

^a^Pearson chi-square

### Participants’ Satisfaction

All of the 70 hill-tribe mothers/caretakers were assessed for their satisfaction with being interviewed by a smartphone, customized-language, voice-based questionnaire survey after they finished the data collection process. The percentages of those who scored ≥80% in each term of satisfaction were: 57% (40/70) for ease of answering using the voiced questionnaire, 69% (48/70) for understanding questions clearly, 71% (50/70) for convenience of responding to questions, and 66% (46/70) for reduced time for data collection (Table 3). Many of the hill-tribe mothers/caretakers had a high level of satisfaction toward the voice-based, questionnaire survey method; they expressed their approval for mobile technology being used for surveys. This was despite a few minor problems during data collection (eg, the system halted for a few seconds, or a poor quality picture was captured by the VHVs).

**Table 3:** Participant’s satisfaction toward the smartphone questionnaire survey (N=70).

Satisfaction toward voice QNN survey		n (%)
**Ease of answering using the voiced questionnaire**		
	≥80% (4-5 score)	40 (57)
	≥50% (2-3 score)	28 (40)
	<50% (1-2 score)	1 (1)
	Missing	1 (1)
**Understanding questions clearly**		
	≥80% (4-5 score)	48 (69)
	≥50% (2-3 score)	20 (29)
	<50% (1-2 score)	1 (1)
	Missing	1 (1)
**Convenience of responding to questions**		
	≥80% (4-5 score)	50 (71)
	≥50% (2-3 score)	19 (27)
	<50% (1-2 score)	-
	Missing	1 (1)
**Reduce time for data collection**		
	≥80% (4-5 score)	46 (66)
	≥50% (2-3 score)	22 (31)
	<50% (1-2 score)	1 (1)
	Missing	1 (1)

## Discussion

### Principal Findings

The technical challenge of applying novel ideas to develop customized-language, voice-based questionnaire functionalities on smartphones for data collection was manageable. Using a smartphone tablet for surveys in the field does not require a telephone signal or wireless network to synchronize data onto a server database. In this study, signals varied, as villages were located in remote highland areas, thus survey activities were conducted off-line and the data were synchronized when the telephone signals or wireless networks became available. This study confirms that data collection via smartphone can be an alternative method to paper-based questionnaires, even in remote/rural areas without the constraint of signal availability [5,9,18]. In this study, though the survey data were transmitted directly from the study tablets to the secured database server at the central office, there was no data encryption mechanism while transferring information to the server. This feature will be implemented in future design.

The results of this study suggest that novel functionalities of picture capturing, and voiced-questions on smartphones can produce data quality, in terms of data completeness, which affect the validity and reliability of the study outcomes. This supports the findings of other data-management studies [19,20]. The same result was found in another study in Thailand [21], in which mobile-phone devices were used to improve antenatal care (ANC) and EPI services in border areas. It was suggested that cell phones developed and integrated into the health care system could be used successfully, especially in low-resource settings [21]. Many previous studies reported the use of handheld computers (PDAs) for data collection [1,4,18,22], but a few revealed that they encountered numerous instances of missing data caused by technical difficulties, or from problems with loss or theft of the devices. Despite such problems, mobile technology use in data collection remains a feasible, acceptable, and preferred method, because it can produce more complete data than the classic paper-based method. Many researchers conducting studies using PDAs [4,22] suggested the use of other hardware solutions, such as tablet smartphones or cell phones, similar to what was done in this study. However, generalizing the result of this study in terms of data completeness could be limited by the cross-over design and small sample size. Even though there was a washout period between the two data-collection methods, the participants may still remember the 55 KAP questions asked using the first method; thus, the comparison of the methods measured data completeness rather than correct answers of the KAP. The focus of this study was to compare data quality between the two methods for the completeness, rather than correctness, of the answers.

Picture capturing for secondary data entry functionality on smartphones in this study demonstrates the successful application of new technology to collect data, especially when collecting data from ethnic minorities who speak different languages. If the data were to be collected on paper-based questionnaire, there could be two chances of transcription errors, from the mothers’ interview or booklet extraction to paper questionnaire and then from paper questionnaire to computer database. In contrast, there should be fewer data transcription errors when using picture-capturing secondary data due to cutting out one step of the data collection process; data are entered directly from the picture of data to the computer database. With the purpose to prove the concept of using picture capturing for secondary data, the current system has not yet designed for the feature of double-data entry to cross-checking data validity. The double-data entry function should be considered in future electronic questionnaire survey design. These features represent how technology can be used as a support tool to facilitate or enhance the work of interviewers and to increase efficiency of survey data collection with the complexity of automated interviewing systems and instruments [12,13].

In this study the use of the smartphone, voice-based questionnaire to collect data on KAP regarding EPI, compared with the paper-based questionnaire, has shown that the data collected was more complete, as the program was designed to ask the questions in sequence automatically. As found in this study, there were incomplete answers when using the paper-based data collection method, but none when using the smartphone, voiced-questionnaire survey method. This may be due to the fact that when performing data collection on paper questionnaires some respondents and/or the interviewers unintentionally skipped a required question, and a few respondents decided not to answer some questions. In contrast, the programming on smartphone survey does not allow the respondents to skip any required question(s). This finding suggests for future design of electronic questionnaires to be more flexible allowing respondents who may want to skip some questions. Thus, the result was similar to the findings of household surveys in South Africa that use mobile phones as data-collection instruments [4,5,23]. Furthermore, previous studies showed that data recording, transferring, and entry mistakes were not found when electronic devices (PDAs, mobile phones, tablets) were used for data collection [8,24,25].

Not only was the benefit of data completeness with the smartphone, voice-based questionnaire confirmed, but the results of this study also suggest that using the smartphone, voice-based questionnaire surveys reduced the time consumed for data collection. This study’s results were similar to the findings of other studies that reported time spent in data collection using mobile technology [3,26-28]. Moreover, the study showed the time consumed during the KAP survey in using the smartphone, voice-based questionnaires that customized to the participant’s own dialect resulted in substantial time saving over paper-based questionnaires. However, the limitation for the use of smartphone, voice-based questionnaires in this study was that none of the voiced-questions were open-ended. With existing technology, collecting open-ended voice answering for the voiced-questions can be done, but that will require programming and adapting the voice recognition solution. If open-ended voice questions are needed, one should plan their data-collection tool with such system functionality. However, as stated in previous studies, when planning to collect data electronically one should consider the appropriateness of methods to be used in accordance with study design, types of data collection, and characteristics of study participants [7,29].

A previous study was conducted in clinical research to compare users’ satisfaction in using two devices for electronic data collection, laptops and handheld computers [25]. It was reported that users found the laptops easier, faster, and more satisfying to use than the handheld computers. This is similar to the findings of a study in China on using handheld data collection tools [22]. Though some technical problems occurred during the data collection process, it remained a feasible, acceptable, and preferred method by Chinese interviewers. In previous studies conducted to assess the acceptability and feasibility of using a mobile phone application for health care delivery among health care workers, conflicting results were reported. The health care workers expressed their positive perceptions toward mHealth, but poor uptake in their actual practice was demonstrated [30]. The survey in this study was conducted as part of the routine home visit by the community health providers (VHVs) who work closely with the village, thus they reported that the use of smartphones could enhance their routine work for health care service in EPI coverage assessment and can be used to educate hill-tribe mothers/caretakers when data collection was finished toward EPI vaccines. Besides interviewer’s satisfaction, this study also assessed satisfaction among participants for data collection with smartphone questionnaires. The results on users’ satisfaction of smartphone-voiced questionnaire surveys in this study confirmed that the underserved, hard to reach, and mostly illiterate users in the remote areas accepted the technology. The questions about technology acceptance used in this study suggest that high percentages of hill-tribe villagers thought that the customized-language, voice-based questionnaire method for data collection made them understand the question more and enabled them to answer accordingly. The survey of VHVs' satisfaction and their overall opinions could be used for improving the design of future electronic questionnaire survey.

One of the limitations of this study was that it was a small-scale survey, thus the costs for conducting the smartphone-questionnaire surveys were higher than the paper-based questionnaires. However, the costs of both data-collection methods would be similar if the study would be conducted on a larger scale. Both methods have different costs according to study scale; however, smartphone questionnaires would still be a feasible alternative for collecting field data even in a low-resource setting. [11,18].

The strength of this study was the data-collection tool, which was developed into a smartphone questionnaire using voiced questions to ask all participants in their dialect languages. As participants of this study speak different languages, and have varied beliefs and cultures, developing and implementing the tool was a challenge. Many languages do not have writing systems, thus most studies conducted among hill-tribe people often used structured, paper-based questionnaires with interviewers and/or translators to collect data [31-34]. This classic method might affect the validity and reliability of data because the way that interviewers/translators pose or translate the questions might induce all participants to answer in a certain direction, and even when asking the same question to different participants the interviewers/translators may use different words/meanings. In developing the smartphone, voice-based questionnaire survey in this study, all questions were first translated and recorded as electronic files in the 6 hill-tribe languages (Karen, Akha, Mien/Yao, Lisu, Lahu, and Yunnan Chinese, as well as Thai), then the translated questions were cross-checked for clear and correct definitions of translation before piloting the tool for the actual local people who speak that language in the field. The final version was used in the study after a few revisions. The results of this study suggest that mobile devices with customized-language, voice-based feasibility would have a potential benefit in minimizing information bias.

### Conclusions

This is the first study to present novel features on using smartphones for survey questionnaires with image-capture and voiced-interrogation functionality. The mobile device can be effectively used for photographing secondary data and collecting primary data with a customized-language and voiced-questionnaire survey. This study has been conducted as a proof-of-concept that smartphone, customized-language, voice-based questionnaire surveys can be successfully used for data collection, especially for the study involving people speaking multiple languages in the study setting. The benefit of using mobile technology to collect data can be observed in terms of reduced time spent in data collection and data entry processes, and minimizing or eliminating missing data when collecting data in the field. Moreover, this study suggests that both interviewers and participants accepted and preferred the technology being used in terms of its ease-of-use and usefulness of the device functionalities (dropdown choices, picture capturing, and voiced questions). There has been an increasing trend in using mobile technology in health care services and health research in the past decade, and further studies can be done, including, but not limited to, the use of several potential beneficial features of mobile technology applications in survey research, including character/voice recognition, geospacing/temporal data management, and animation questions.

## Abbreviations

ANC: antenatal care
EPI: expanded program on immunization
KAP: knowledge attitude practice
MCHH: Maternal and Child Health Handbook
PDAs: personal digital assistants
TAM: Technology Acceptance Model
VHVs: village health volunteers
